# 1,6-Bis(*p*-tol­yloxy)hexa­ne

**DOI:** 10.1107/S1600536814009222

**Published:** 2014-04-30

**Authors:** XiuQin Zhang, Jian Xiao, Wei-Wei Xue, Qiang Chen, Kai Wang

**Affiliations:** a High Technology Research Institute of Nanjing University, Changzhou 213162, Jiangsu, People’s Republic of China; b School of Petrochemical Engineering, Changzhou University, Changzhou 213164, Jiangsu, People’s Republic of China

## Abstract

The title compound, C_20_H_26_O_2_, crystallized with one half-mol­ecule in the asymmetric unit. The whole mol­ecule is generated by inversion symmetry, with the center of inversion being situated at the mid-point of the central –CH_2_—CH_2_- bond of the bridging hexane chain. In the crystal, mol­ecules stack in columns along the *b* axis. C—H⋯π inter­actions are present within the columns.

## Related literature   

For the properties and synthesis of the title compound, see: Saito *et al.* (1988 ▶). For bond-length data, see: Allen *et al.* (1987 ▶).
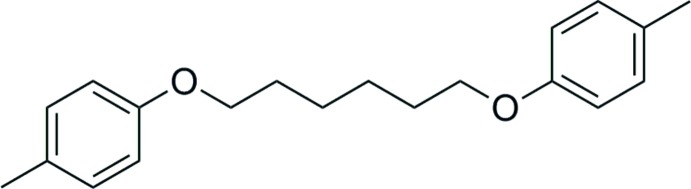


## Experimental   

### 

#### Crystal data  

C_20_H_26_O_2_
*M* *_r_* = 298.41
Monoclinic, 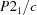

*a* = 18.932 (12) Å
*b* = 7.327 (4) Å
*c* = 6.352 (4) Å
β = 91.000 (13)°
*V* = 881.0 (9) Å^3^
*Z* = 2
Mo *K*α radiation
μ = 0.07 mm^−1^
*T* = 293 K
0.25 × 0.20 × 0.18 mm

#### Data collection  

Enraf–Nonius CAD-4 diffractometer
Absorption correction: ψ scan (North *et al.*, 1968 ▶) *T*_min_ = 0.983, *T*_max_ = 0.987
4572 measured reflections
1544 independent reflections
963 reflections with *I* > 2σ(*I*)
*R*_int_ = 0.117
3 standard reflections every 200 reflections intensity decay: 1%

#### Refinement  

*R*[*F* ^2^ > 2σ(*F* ^2^)] = 0.077
*wR*(*F* ^2^) = 0.316
*S* = 1.10
1544 reflections
101 parameters
H-atom parameters constrained
Δρ_max_ = 0.36 e Å^−3^
Δρ_min_ = −0.32 e Å^−3^

### 

Data collection: *CAD-4 Software* (Enraf–Nonius, 1985 ▶); cell refinement: *CAD-4 Software*; data reduction: *XCAD4* (Harms & Wocadlo,1995 ▶); program(s) used to solve structure: *SHELXS97* (Sheldrick, 2008 ▶); program(s) used to refine structure: *SHELXL97* (Sheldrick, 2008 ▶); molecular graphics: *SHELXTL* (Sheldrick, 2008 ▶); software used to prepare material for publication: *SHELXTL*.

## Supplementary Material

Crystal structure: contains datablock(s) I, zxq. DOI: 10.1107/S1600536814009222/su2725sup1.cif

Structure factors: contains datablock(s) I. DOI: 10.1107/S1600536814009222/su2725Isup2.hkl

Supporting information file. DOI: 10.1107/S1600536814009222/su2725Isup3.cml

CCDC reference: 999158

Additional supporting information:  crystallographic information; 3D view; checkCIF report

## Figures and Tables

**Table 1:** Hydrogen-bond geometry (Å, °) *Cg* is the centroid of the C2–C7 benzene ring.

*D*—H⋯*A*	*D*—H	H⋯*A*	*D*⋯*A*	*D*—H⋯*A*
C4—H4⋯*Cg* ^i^	0.93	2.95	3.696 (4)	138
C7—H7⋯*Cg* ^ii^	0.93	2.84	3.572 (4)	137

Symmetry codes: (i) 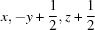; (ii) 
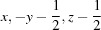
.

**Fig. 1.**
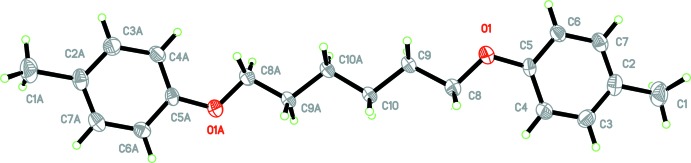
The molecular structure of the title molecule, with atom labelling. Displacement ellipsoids are drawn at the 50% probability level.

**Fig. 2.**
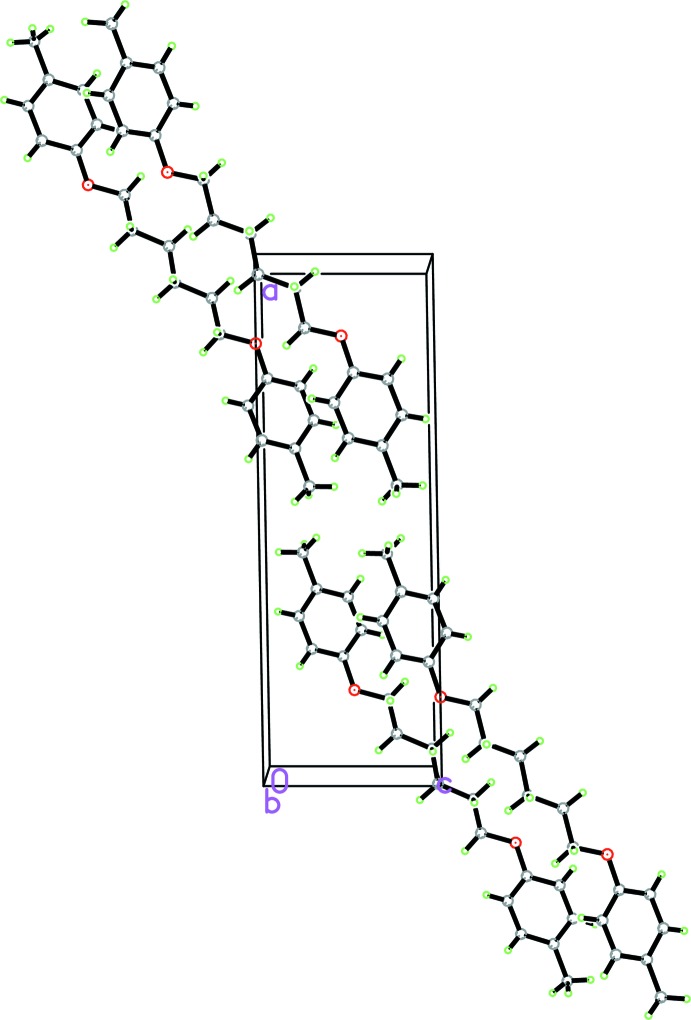
A view along the b axis of the crystal packing of the title compound.

## supplementary crystallographic information

### 1. Comment

The title compound is used as a sensitizer for thermal recording materials, polyester-resin monomers and fire-resistant materials (Saito *et al.*, 1988).

The molecular structure of the title compound is shown in Fig. 1. The bond lengths (Allen *et al.* (1987) and angles are within normal ranges. It crystallized with half a molecule in the asymmetric unit. The whole molecule is generated by inversion symmetry with the center of inversion being situated at the center of the C10—C10^i^ bond of the bridging hexane chain [symmetry code: (i) -*x*, -*y*, -*z* + 2].

In the crystal, there are no intermolecular hydrogen bonds present (Fig. 2). The molecules stack in columns along the *b* axis and within the columns there are C—H···π interactions present (Table 1).

### 2. Experimental

The title compound was prepared by the reported procedure (Saito *et al.*, 1988). Anhydrous potassium carbonate (6.2 g, 45 mmol) was added to a solution of 1,6-dibromohexane (2.5 g, 10.25 mmol) and 4-methoxyphenol (3.18 g, 25.6 mmol) in acetonitrile (100 ml). The mixture was stirred overnight at 338 K, and then filtered and the filtrate evaporated under reduced pressure. The residue was subjected to flash chromatography on silica gel, eluting with (10:1/petroleum ether:ethyl acetate) to give the title compound (Yield 2.13 g). Colourless block-like crystals of the title compound were obtained by slow evaporation of a solution in ethanol (20 ml), after ca. 7 days.

### 3. Refinement

All the H atoms were positioned geometrically and constrained to ride on their parent atoms: C—H = 0.93 - 0.97 Å with *U*_iso_(H) = 1.5*U*_eq_(*C*-methyl) and = 1.2*U*_eq_(C) for other H atoms.

### Crystal data

**Table:**

C_20_H_26_O_2_	*F*(000) = 324
*M**_r_* = 298.41	*D*_x_ = 1.125 Mg m^−^^3^
Monoclinic, *P*2_1_/*c*	Mo *K*α radiation, λ = 0.71073 Å
Hall symbol: -P 2ybc	Cell parameters from 1071 reflections
*a* = 18.932 (12) Å	θ = 2.2–23.2°
*b* = 7.327 (4) Å	µ = 0.07 mm^−^^1^
*c* = 6.352 (4) Å	*T* = 293 K
β = 91.000 (13)°	Block, colourless
*V* = 881.0 (9) Å^3^	0.25 × 0.20 × 0.18 mm
*Z* = 2	

### Data collection

**Table:**

Enraf–Nonius CAD-4 diffractometer	963 reflections with *I* > 2σ(*I*)
Radiation source: fine-focus sealed tube	*R*_int_ = 0.117
Graphite monochromator	θ_max_ = 25.0°, θ_min_ = 1.1°
ω/2θ scans	*h* = −22→21
Absorption correction: ψ scan (North *et al.*, 1968)	*k* = −5→8
*T*_min_ = 0.983, *T*_max_ = 0.987	*l* = −7→7
4572 measured reflections	3 standard reflections every 200 reflections
1544 independent reflections	intensity decay: 1%

### Refinement

**Table:**

Refinement on *F*^2^	Primary atom site location: structure-invariant direct methods
Least-squares matrix: full	Secondary atom site location: difference Fourier map
*R*[*F*^2^ > 2σ(*F*^2^)] = 0.077	Hydrogen site location: inferred from neighbouring sites
*wR*(*F*^2^) = 0.316	H-atom parameters constrained
*S* = 1.10	*w* = 1/[σ^2^(*F*_o_^2^) + (0.2*P*)^2^] where *P* = (*F*_o_^2^ + 2*F*_c_^2^)/3
1544 reflections	(Δ/σ)_max_ < 0.001
101 parameters	Δρ_max_ = 0.36 e Å^−^^3^
0 restraints	Δρ_min_ = −0.32 e Å^−^^3^

### Special details

**Table:**

Geometry. All e.s.d.'s (except the e.s.d. in the dihedral angle between two l.s. planes) are estimated using the full covariance matrix. The cell e.s.d.'s are taken into account individually in the estimation of e.s.d.'s in distances, angles and torsion angles; correlations between e.s.d.'s in cell parameters are only used when they are defined by crystal symmetry. An approximate (isotropic) treatment of cell e.s.d.'s is used for estimating e.s.d.'s involving l.s. planes.
Refinement. Refinement of *F*^2^ against ALL reflections. The weighted *R*-factor *wR* and goodness of fit *S* are based on *F*^2^, conventional *R*-factors *R* are based on *F*, with *F* set to zero for negative *F*^2^. The threshold expression of *F*^2^ > σ(*F*^2^) is used only for calculating *R*-factors(gt) *etc*. and is not relevant to the choice of reflections for refinement. *R*-factors based on *F*^2^ are statistically about twice as large as those based on *F*, and *R*- factors based on ALL data will be even larger.

### Fractional atomic coordinates and isotropic or equivalent isotropic displacement parameters (Å^2^)

**Table:**

	*x*	*y*	*z*	*U*_iso_*/*U*_eq_	
O1	0.15451 (11)	−0.0150 (4)	0.5211 (3)	0.0524 (8)	
C1	0.4342 (2)	−0.0202 (7)	0.2221 (7)	0.0812 (15)	
H1A	0.4508	−0.1439	0.2302	0.122*	
H1B	0.4644	0.0567	0.3067	0.122*	
H1C	0.4349	0.0203	0.0784	0.122*	
C2	0.36013 (17)	−0.0103 (5)	0.3018 (6)	0.0535 (10)	
C3	0.34523 (17)	0.0740 (5)	0.4910 (6)	0.0555 (10)	
H3	0.3819	0.1277	0.5682	0.067*	
C4	0.27798 (16)	0.0806 (5)	0.5681 (5)	0.0497 (10)	
H4	0.2691	0.1409	0.6937	0.060*	
C5	0.22302 (16)	−0.0041 (4)	0.4560 (5)	0.0400 (9)	
C6	0.23675 (16)	−0.0871 (5)	0.2660 (4)	0.0439 (9)	
H6	0.2002	−0.1416	0.1892	0.053*	
C7	0.30380 (17)	−0.0892 (5)	0.1906 (5)	0.0502 (10)	
H7	0.3121	−0.1448	0.0618	0.060*	
C8	0.13751 (15)	0.0427 (5)	0.7259 (5)	0.0456 (9)	
H8A	0.1378	0.1749	0.7336	0.055*	
H8B	0.1719	−0.0042	0.8273	0.055*	
C9	0.06519 (15)	−0.0294 (5)	0.7732 (4)	0.0449 (9)	
H9A	0.0669	−0.1617	0.7745	0.054*	
H9B	0.0327	0.0074	0.6612	0.054*	
C10	0.03691 (14)	0.0366 (5)	0.9817 (4)	0.0403 (9)	
H10A	0.0358	0.1690	0.9826	0.048*	
H10B	0.0682	−0.0032	1.0951	0.048*	

### Atomic displacement parameters (Å^2^)

**Table:**

	*U*^11^	*U*^22^	*U*^33^	*U*^12^	*U*^13^	*U*^23^
O1	0.0479 (16)	0.0683 (18)	0.0413 (14)	−0.0032 (10)	0.0101 (10)	−0.0063 (11)
C1	0.064 (3)	0.093 (4)	0.088 (3)	0.002 (2)	0.034 (2)	−0.005 (3)
C2	0.053 (2)	0.043 (2)	0.065 (2)	0.0025 (15)	0.0202 (17)	0.0055 (17)
C3	0.051 (2)	0.051 (2)	0.065 (2)	−0.0086 (16)	0.0069 (16)	−0.0079 (18)
C4	0.055 (2)	0.049 (2)	0.0456 (18)	−0.0020 (15)	0.0089 (15)	−0.0109 (16)
C5	0.0429 (18)	0.0390 (19)	0.0385 (18)	0.0012 (13)	0.0136 (13)	0.0055 (13)
C6	0.0538 (19)	0.043 (2)	0.0346 (16)	−0.0022 (14)	0.0030 (13)	0.0022 (14)
C7	0.065 (2)	0.043 (2)	0.0433 (18)	0.0040 (16)	0.0137 (15)	−0.0009 (15)
C8	0.0469 (19)	0.049 (2)	0.0411 (18)	0.0042 (14)	0.0062 (14)	−0.0022 (14)
C9	0.0475 (19)	0.053 (2)	0.0341 (17)	−0.0005 (14)	0.0093 (13)	−0.0078 (14)
C10	0.0457 (19)	0.046 (2)	0.0297 (16)	0.0024 (13)	0.0059 (13)	−0.0025 (12)

### Geometric parameters (Å, º)

**Table:**

O1—C5	1.371 (3)	C6—C7	1.365 (4)
O1—C8	1.410 (4)	C6—H6	0.9300
C1—C2	1.502 (4)	C7—H7	0.9300
C1—H1A	0.9600	C8—C9	1.503 (4)
C1—H1B	0.9600	C8—H8A	0.9700
C1—H1C	0.9600	C8—H8B	0.9700
C2—C3	1.385 (5)	C9—C10	1.516 (4)
C2—C7	1.394 (5)	C9—H9A	0.9700
C3—C4	1.373 (4)	C9—H9B	0.9700
C3—H3	0.9300	C10—C10^i^	1.519 (5)
C4—C5	1.396 (5)	C10—H10A	0.9700
C4—H4	0.9300	C10—H10B	0.9700
C5—C6	1.380 (4)		
			
C5—O1—C8	119.6 (2)	C6—C7—C2	121.7 (3)
C2—C1—H1A	109.5	C6—C7—H7	119.2
C2—C1—H1B	109.5	C2—C7—H7	119.2
H1A—C1—H1B	109.5	O1—C8—C9	107.6 (3)
C2—C1—H1C	109.5	O1—C8—H8A	110.2
H1A—C1—H1C	109.5	C9—C8—H8A	110.2
H1B—C1—H1C	109.5	O1—C8—H8B	110.2
C3—C2—C7	117.3 (3)	C9—C8—H8B	110.2
C3—C2—C1	121.3 (4)	H8A—C8—H8B	108.5
C7—C2—C1	121.3 (3)	C8—C9—C10	113.5 (3)
C4—C3—C2	122.0 (3)	C8—C9—H9A	108.9
C4—C3—H3	119.0	C10—C9—H9A	108.9
C2—C3—H3	119.0	C8—C9—H9B	108.9
C3—C4—C5	119.3 (3)	C10—C9—H9B	108.9
C3—C4—H4	120.3	H9A—C9—H9B	107.7
C5—C4—H4	120.3	C9—C10—C10^i^	111.2 (3)
O1—C5—C6	115.6 (3)	C9—C10—H10A	109.4
O1—C5—C4	124.9 (3)	C10^i^—C10—H10A	109.4
C6—C5—C4	119.5 (3)	C9—C10—H10B	109.4
C7—C6—C5	120.2 (3)	C10^i^—C10—H10B	109.4
C7—C6—H6	119.9	H10A—C10—H10B	108.0
C5—C6—H6	119.9		
			
C7—C2—C3—C4	0.0 (5)	C4—C5—C6—C7	1.3 (5)
C1—C2—C3—C4	−178.6 (4)	C5—C6—C7—C2	0.6 (5)
C2—C3—C4—C5	1.8 (5)	C3—C2—C7—C6	−1.2 (5)
C8—O1—C5—C6	171.1 (3)	C1—C2—C7—C6	177.4 (4)
C8—O1—C5—C4	−7.9 (5)	C5—O1—C8—C9	−165.3 (3)
C3—C4—C5—O1	176.6 (3)	O1—C8—C9—C10	−174.7 (3)
C3—C4—C5—C6	−2.4 (5)	C8—C9—C10—C10^i^	178.6 (3)
O1—C5—C6—C7	−177.8 (3)		

Symmetry code: (i) −*x*, −*y*, −*z*+2.

### Hydrogen-bond geometry (Å, º)

Cg is the centroid of the C2–C7 benzene ring.

**Table:**

*D*—H···*A*	*D*—H	H···*A*	*D*···*A*	*D*—H···*A*
C4—H4···*Cg*^ii^	0.93	2.95	3.696 (4)	138
C7—H7···*Cg*^iii^	0.93	2.84	3.572 (4)	137

Symmetry codes: (ii) *x*, −*y*+1/2, *z*+1/2; (iii) *x*, −*y*−1/2, *z*−1/2.
